# NSD1 mutations by HPV status in head and neck cancer: differences in survival and response to DNA-damaging agents

**DOI:** 10.1186/s41199-019-0042-3

**Published:** 2019-07-08

**Authors:** Cassie Pan, Said Izreig, Wendell G. Yarbrough, Natalia Issaeva

**Affiliations:** 1 Department of Surgery, Division of Otolaryngology, Yale University, New Haven, CT USA; 2 Department of Otolaryngology/Head and Neck Surgery, The University of North Carolina at Chapel Hill, 170 Manning Drive, Campus Box 7070, Chapel Hill, NC 27599-7070 USA; 3 Lineberger Comprehensive Cancer Center, University of North Carolina at Chapel Hill, Chapel Hill, NC USA

**Keywords:** Head and neck cancer, HPV, NSD1, Survival, Treatment

## Abstract

**Background:**

Compared to HPV-negative head and neck squamous cell carcinomas (HNSCCs), HPV-positive HNSCCs are associated with a favorable prognosis in part due to their improved treatment sensitivity. Inactivating mutations in NSD1 were shown to be a favorable prognostic biomarker in laryngeal cancers. Here, we characterize NSD1 mutations from the expanded The Cancer Genome Atlas (TCGA) HNSCC cohort (n = 522) and examine their prognostic implications based on HPV status of the tumor. We also begin to examine if NSD1 regulates response to platinum-based drugs and other DNA-damaging agents.

**Methods:**

TCGA HNSCC samples were segregated by HPV and NSD1 mutations using cBioPortal and patient survival was determined. Pathogenicity of mutations was predicted using UMD-Predictor. NSD1-depleted cell lines were established by transfection with control or shRNAs against NSD1, followed by puromycin selection, and confirmed by qRT-PCR. Cell sensitivity to DNA damaging agents was assessed using short-term proliferation and long-term clonogenic survival assays.

**Results:**

Among 457 HPV(−) tumors, 13% contained alterations in the NSD1 gene. The majority (61.3%) of NSD1 gene alterations in HPV(−) specimens were truncating mutations within or before the enzymatic SET domain. The remaining alterations included homozygous gene deletions (6.7%), missense point mutations (30.7%) and inframe deletions (1.3%). UMD-Predictor categorized 18 of 23 missense point mutations as pathogenic. For HPV(+) HNSCC (n = 65), 6 NSD1 mutations, comprised of two truncating (33%) and 4 missense point (66%) mutations, were identified. Three of the 4 missense point mutations were predicted to be pathogenic or probably pathogenic by UMD-Predictor. Kaplan-Meier survival analysis determined significantly improved survival of HPV(−) HNSCC patients whose tumors harbored NSD1 gene alterations, as compared to patients with wild-type NSD1 tumors. Interestingly, the survival effect of NSD1 mutations observed in HPV-negative HNSCC was reversed in HPV(+) tumors. Proliferation and clonogenic survival of two HPV(−) cell lines stably expressing control or NSD1 shRNAs showed that NSD1-depleted cells were more sensitive to cisplatin and carboplatin, but not to other DNA damaging drugs.

**Conclusions:**

Genetic alterations in NSD1 hold potential as novel prognostic biomarkers in HPV(−) head and neck cancers. NSD1 mutations in HPV(+) cancers may also play a prognostic role, although this effect must be examined in a larger cohort. NSD1 downregulation results in improved sensitivity to cisplatin and carboplatin, but not to other DNA-damaging agents, in epithelial cells. Increased sensitivity to platinum-based chemotherapy agents associated with NSD1 depletion may contribute to improved survival in HPV(−) HNSCCs. Further studies are needed to determine mechanisms through which NSD1 protects HPV(−) HNSCC cells from platinum-based therapy, as well as confirmation of NSD1 effect in HPV(+) HNSCC.

## Background

Head and neck squamous cell carcinomas (HNSCCs) comprise a diverse group of tumors, which are classified into various anatomical sites (e.g. oral cavity, oropharynx, hypopharynx, larynx, and nasopharynx), with unique epidemiology, anatomy, clinical behavior, and association with human papilloma virus (HPV) or Epstein-Barr Virus infection [1, 2]. HPV-negative (HPV(−)) head and neck cancers are strongly associated with tobacco and alcohol use and are most commonly found in the oral cavity and larynx [3]. Rates of HPV(−) cancers have declined in recent decades with the decreasing rates of smoking, but during this same period, rates of oropharyngeal cancers continued to increase [3–7]. The majority of oropharyngeal squamous cell carcinomas (OPSCCs) are associated with HPV and occur in younger patients with minimal or no tobacco exposure [8–13]. HPV-positive (HPV(+)) OPSCCs carry a favorable prognosis compared to HPV-negative cancers. Five-year survival rates for patients with HPV(+) OPSCC are 60–90%, versus survival rates of 20–25% among patients with HPV(−) OPSCCs (survival rates for HPV(−) tumors of all sites is 65%) [3]. The improved survival of patients with HPV(+) tumors can in part be attributed to their remarkable treatment sensitivity, as HPV(+) tumors have been shown to respond better to chemotherapy and radiation than HPV(−) tumors [13, 14].

For HPV(−) cancers, which are significantly more deadly than HPV(+) cancers, understanding their molecular diversity is critical to developing and selecting appropriate treatments to improve mortality. Comprehensive genomic profiling of head and neck squamous cell carcinomas by The Cancer Genome Atlas (TCGA) has identified a distinct subgroup of HPV(−) cancers with inactivating mutations in the nuclear receptor-binding SET domain protein 1 (NSD1), a methyltransferase and chromatin modifier [15, 16]. In a recent study, loss-of-function mutations in NSD1 were shown to be a favorable prognostic biomarker in laryngeal cancers, which are almost uniformly HPV(−) and strongly associated with tobacco and alcohol use [17]. However, no studies have examined the differences of NSD1 mutations between HPV-positive and HPV-negative head and neck cancers. Certainly, much remains to be elucidated regarding the role of NSD1 in oncogenesis, and knowledge of NSD1 direct targets is currently very limited. As for the distinct subgroup within HPV(−) head and neck cancers with inactivating NSD1 mutations, the clinical implications of this genetic alteration have yet to be fully explored [18].

In this study, we characterize NSD1 mutations in HPV(+) and HPV(−) cohorts from the expanded TCGA HNSCC dataset and examine prognostic implications of NSD1 mutations in the TCGA dataset. Finally, we investigate response of NSD1-depleted cells to platinum-based chemotherapy drugs and a variety of agents that have different mechanisms of DNA damage.

## Methods

### NSD1 mutations analysis in TCGA cohort

**Fig. 1:**
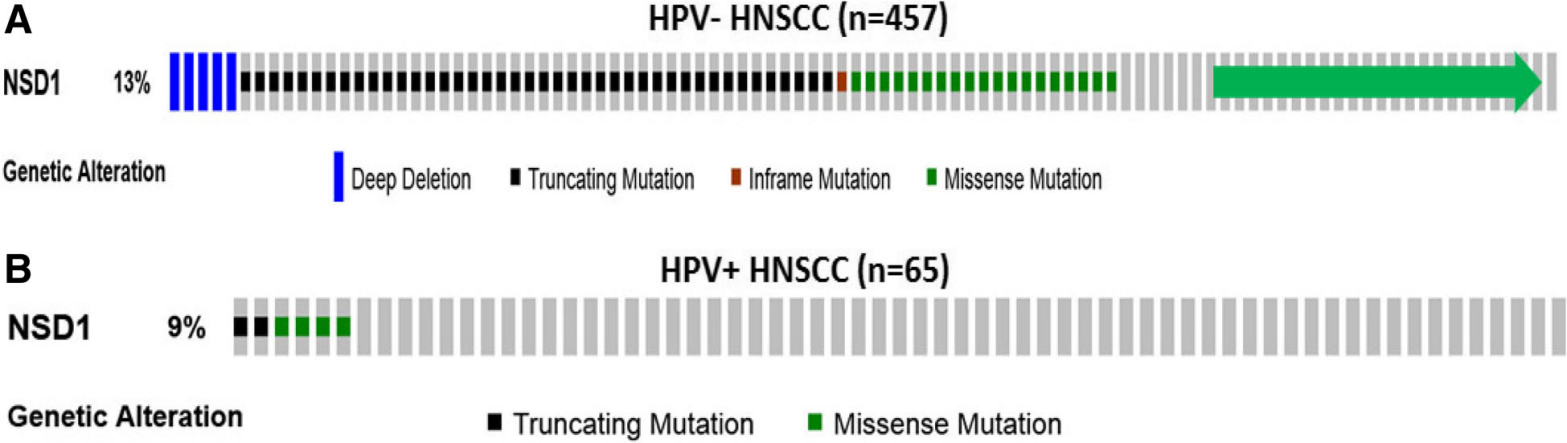
NSD1 mutations in the expanded TCGA HNSCC cohort. Alterations in NSD1 gene found in (**a**) HPV-negative (n = 457) and (**b**) HPV-positive (n = 65) HNSCC. Each tumor is represented as a column. The green arrow indicates tumors without alterations that were cut to fit the figure. Adapted from cBioPortal.org [41, 42]

**Fig. 2:**
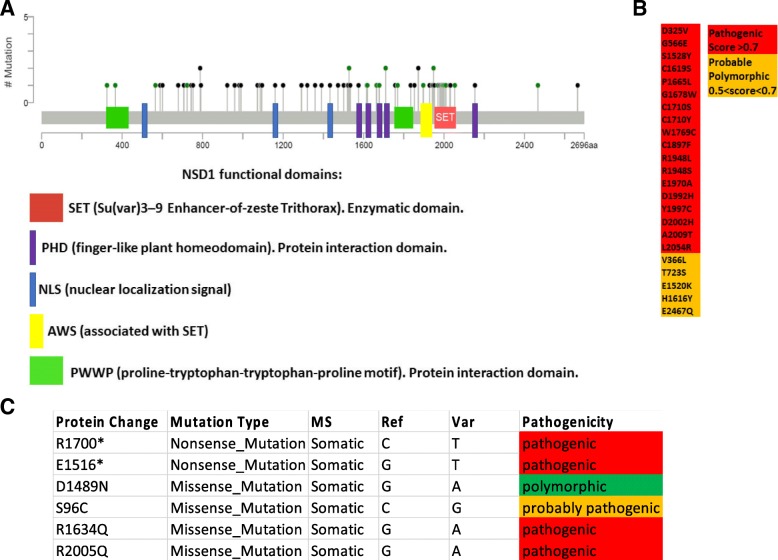
NSD1 mutations in HPV(−) and HPV(+) HNSCC are inactivating mutations. (**a**) Schematic representation of missense point (green) and truncating (black) mutations in NSD1 found in HPV-negative HNSCC; functional NSD1 domains are indicated in colors. Adapted from www.cBioPortal.org [41, 42]. **b** Pathogenicity of HPV(−) NSD1 missense point mutations. (**c**) Pathogenicity of HPV(+) NSD1 mutations. Pathogenicity determined with UMD-predictor [21]

TCGA HNSCC database was mined for HPV(−) and HPV(+) samples (HPV(+) defined as ≥100 HPV reads per million human reads in whole-genome sequencing. Of the initial group of HPV(+) cases detected, those with p53 mutations were redesignated as HPV(−) cases, as the HPV DNA detected was likely not a driver of carcinogenesis. NSD1 mutations were identified with cBioPortal (available at: cbioportal.org). Figure 1 and Fig. 2 A were downloaded and adapted from the portal.

### Kaplan-Meier survival analysis

Kaplan-Meier curves illustrating the survival of patients with HPV(−) and HPV(+) HNSCC with and without mutations in NSD1 were produced and downloaded from cBioPortal, and the log-rank (Mantel-Cox) test was used for statistical analyses.

### Cell lines, constructs and chemicals

FaDu (human HPV(−) hypopharyngeal HNSCC) and SCC35 (human HPV(−) pharyngeal HNSCC) cells were cultured in DMEM/F12 medium supplemented with 0.4 μg/mL hydrocortisone. HaCat (spontaneously transformed non-tumorigenic human keratinocyte) cells were grown in DMEM with nonessential amino acids. All media was supplemented with 10% FBS (Invitrogen), 50 μg/mL penicillin, and 50 μg/mL streptomycin (Invitrogen). All cell lines tested negative for Mycoplasma and microsatellites authenticated.

### Establishment of NSD1-depleted cells

FaDu [16], SCC35, and HaCat [19] cells were transfected with control (SHC016) or 2 different shRNAs against NSD1 (TRCN0000238372 and TRCN0000238371) obtained from Sigma. Stably transfected cells were selected using puromycin resistance. Clones were tested for NSD1 knockdown using quantitative RT-PCR with primer pairs listed below; GAPDH was used as a reference gene.

Primer pairs:

**Table:** 

NSD1mF	GGCCATTGCTACCTGAAAGA
NSD1mR	GGAAACCAAGGATTGGGATT
GAPDHF	AGGGCTGCTTTTAACTCTGGT
GAPDHR	CCCCACTTGATTTTGGAGGGA
ERCC5mF	CCTCAGAACAAGGCGAAGAG
ERCC5mR	CCATTCATGGAGCGAATCTT

### RNA extraction and quantitative RT-PCR

Total RNA was extracted by Qiagen RNA extraction kit and cDNA was synthesized using iScript cDNA Synthesis Kit (Bio-Rad) according to the manufacturer’s instructions. Quantitative real-time reverse transcription (qRT-PCR) was done using iQ SYBR Green Supermix (Bio-Rad) and indicated primer pairs on the iCycler iQ Real-Time PCR Detection System (Bio-Rad). Each qRT-PCR reaction was done in duplicate at least, and the ΔΔCt method was used to analyze the data; relative expression (% from GPDH) is presented.

### Short-term proliferation assays

Cells (3000 cells/well) were plated in 96-well plates and treated with increasing doses of cisplatin (0–16 μM), carboplatin (0–20 μg/ml), etoposide (0–25 μM), zeocin (0–480 μg/ml), paclitaxel (0–160 nM), or mirin (0–80 μM) in triplicate. Proliferation was determined 72 h after treatment using the Cell Titer Glo reagent (Promega).

### Long-term clonogenic survival assays

Cells (1000 cells/well) were plated in 6-well plates and treated with carboplatin (0–1 μg/ml for FaDu, 0–2 μg/ml for HaCat) in duplicates. After cell growth for 10 days for HaCat cells and 14 days for FaDu cells, colonies were fixed and stained with methylene blue in methanol for 1 h. Colonies consisting of at least 50 cells were counted.

## Results

### Distinct subgroups of HPV(+) and HPV(−) tumors harbor inactivating NSD1 mutations

**Fig. 3:**
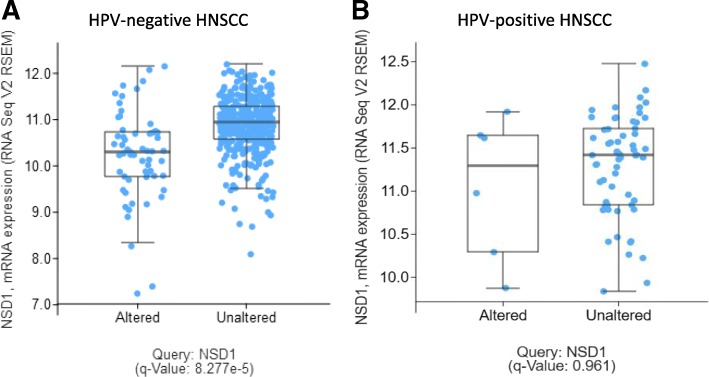
Mutant NSD1 is underexpressed in HPV(−), but not in HPV(+) tumors. Scatterplot showing expression of wild-type and mutant NSD1 in (**a**) HPV(−) tumors and (**b**) HPV(+) tumors. Adapted from cBioPortal.org [41, 42]

In the previously published TCGA HNSCC cohorts (*n* = 273 [15] or n = 421 [20]), NSD1 was identified as a significantly mutated gene. We found NSD1 gene alterations in 13% of HPV-negative (n = 457) and 9% of HPV-positive (n = 65) tumors (Fig. 1). The majority (61.3%) of NSD1 gene alterations in HPV(−) specimens were truncating mutations that occurred within or before the enzymatic SET domain of the NSD1 protein (Fig. 2A). The remaining alterations included homozygous gene deletions (6.7%), missense point mutations (30.7%) and a single in-frame deletion (1.3%). UMD-Predictor assigned 18 out of the 23 missense point mutations as pathogenic (Fig. 2B) [21]. Furthermore, mutant NSD1 was found to be underexpressed in HPV(−) tumors compared to wild-type NSD1 (Fig. 3A). Likewise, of the six HPV(+) NSD1 mutations, which were comprised of truncating (33%) and missense point (66%) mutations (Fig. 1), 3 of the 4 missense point mutations were predicted to be pathogenic or probably pathogenic by UMD-Predictor (Fig. 2C). However, in contrast to HPV(−) tumors, mutant NSD1 was not underexpressed in our limited set (n = 6) of HPV(+) head and neck cancer specimens (Fig. 3B), though this finding must be examined in a larger cohort before any definitive conclusions can be drawn.

### Clinical characteristics of HPV(−) and HPV(+) tumors comparing those with and without NSD1 mutations

Statistical analysis of clinical characteristics of the HPV(−) cohort demonstrated that the NSD1 mutant and wild-type groups did not differ significantly in terms of average age at diagnosis or sex, race, alcohol use, or clinical stage (Table 1). Differences in smoking history were detected, with the cohort harboring NSD1 mutations having higher proportions of current and former smokers compared to those without NSD1 mutations (p < 0.001). Although only a small number of HPV(+) tumors were available for analysis, no differences between the NSD1 mutant and wild-type groups were found in average age at diagnosis, sex, race, smoking history, alcohol use, or stage (Table 2). Thus, NSD1 mutations are not associated with any clinical characteristics, except of smoking status in HPV-negative tumors.

**Table 1:** Clinical characteristics of TCGA HPV(−) cohort by NSD1 mutation status

	Mutated NSD1, n = 60		Wild-type NSD1, *n* = 397		*p*-value*
	n	%	n	%	
Avg. age at diagnosis (years)	60.0		61.6		0.25
Sex					0.16
Female	13	21.7	121	30.5	
Male	47	78.3	276	69.5	
Race					0.59
American Indian or Alaska Native	0	0.0	2	0.5	
Asian	0	0.0	11	2.8	
Black or African American	7	11.7	38	9.6	
White	50	83.3	334	84.1	
Unknown	3	5.0	12	3.0	
Smoking History					< 0.001
Never Smoker	2	3.3	99	24.9	
Current Smoker	32	53.3	129	32.5	
Former Smoker	25	41.7	158	39.8	
Blank	1	1.7	11	2.8	
Alcohol Use					0.37
No	15	25.0	134	33.8	
Yes	44	73.3	254	64.0	
Unknown	1	1.7	9	2.3	
Stage^a^					0.15
Stage I, II	9	15.0	97	24.4	
Stage III	19	31.7	81	20.4	
Stage IV	31	51.7	207	52.1	
Unknown	1	1.7	12	3.0	

*p-value is calculated from chi-square test for categorical variables or Wilcoxon rank-sum test for age (continuous)

^a^Stages assigned using AJCC 7th edition (76%), 6th edition (21%), 5th edition (2%), and 4th edition (< 1%)

**Table 2:** Clinical characteristics of TCGA HPV(+) cohort by NSD1 mutation status

	Mutant NSD1, *n* = 6		Wild-type NSD1, *n* = 59		*p*-value*
	n	%	n	%	
Avg. age at diagnosis (years)	51.8		57.8		0.15
Sex					0.41
Female	0	0.0	6	10.2	
Male	6	100.0	53	89.8	
Race					0.57
Black or African American	0	0.0	3	5.1	
White	6	100.0	56	94.9	
Smoking History Category					0.53
Never Smoker	3	50.0	18	30.5	
Current Smoker	0	0.0	14	23.7	
Former Smoker	3	50.0	26	44.1	
Unknown	0	0.0	1	1.7	
Alcohol Use					0.90
No	1	16.7	13	22.0	
Yes	5	83.3	45	76.3	
Unknown	0	0.0	1	1.7	
Stage^a^					0.65
Stage I, II	1	16.7	11	18.6	
Stage III	0	0.0	7	11.9	
Stage IV	5	83.3	41	69.5	

*p-value is calculated from chi-square test for categorical variables or Wilcoxon rank-sum test for age (continuous)

^a^Stages assigned using AJCC 7th edition (63%), 6th edition (37%)

### NSD1 mutations are enriched in laryngeal HPV(−) tumors

**Fig. 4:**
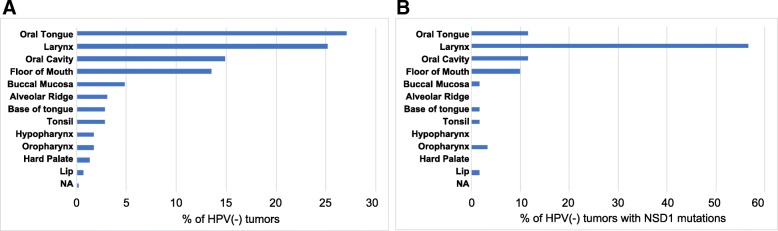
NSD1 mutations are enriched in HPV(−) laryngeal tumors. (**a**) Percentage by subsite of HPV(−) tumors (*n* = 457) in expanded TCGA cohort. (**b**) Percentage of NSD1 mutations (*n* = 60) in HPV(−) tumors, grouped by subsite

Classification of the TCGA HPV(−) cohort by site revealed that laryngeal cancers were overrepresented amongst those with mutant NSD1: 25% of HPV(−) tumors arose in the larynx but almost 60% of HPV(−) tumors with NSD1 mutations occurred in the larynx (Fig. 4). In contrast, oral tongue tumors were underrepresented in the NSD1 mutant cohort, comprising 27% of HPV(−) tumors but only 11% of HPV(−) tumors with NSD1 mutations. In agreement with prior reports [17], NSD1 mutations are enriched in laryngeal HPV(−) tumors.

### NSD1 mutations correlate with improved survival in HPV(−) HNSCC but worse survival in HPV(+) HNSCC

**Fig. 5:**
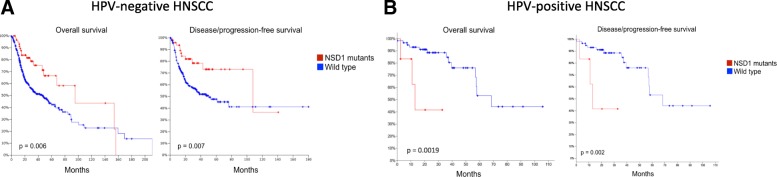
NSD1 mutations correlate with improved survival in HPV(−) HNSCC and worse survival in HPV(+) HNSCC. Kaplan–Meier curves showing survival of patients with (**a**) HPV(−) tumors and (**b**) HPV(+) tumors, with or without alterations in NSD1 gene. Overall survival and disease/progression-free survival are shown

We next performed Kaplan-Meier survival analysis of head and neck patients in the expanded TCGA cohort based on HPV and NSD1 mutation status. Interestingly, patients with HPV-negative HNSCC whose tumors contain alterations in the NSD1 gene (n = 60), showed significantly improved overall (p = 0.006) and disease-free (p = 0.007) survival compared to patients with tumors that harbor wild-type NSD1 (n = 397) (Fig. 5A). Improved survival associated with NSD1 mutations in HPV-negative HNSCC is consistent with previously published work showing that NSD1 mutations are a favorable prognostic biomarker in head and neck tumors [17, 20]. In contrast, survival analysis on HPV(+) tumors with and without NSD1 mutations surprisingly revealed that NSD1 mutations were associated with significantly worse overall (p = 0.0019) and disease/progression-free survival (p = 0.002) (Fig. 5B). These data suggest that NSD1 mutations may have different impact on patients’ survival based on HPV status, although confirmation is needed in a larger HPV-positive HNSCC cohort.

### NSD1 mutations are associated with increased sensitivity to cisplatin

**Fig. 6:**
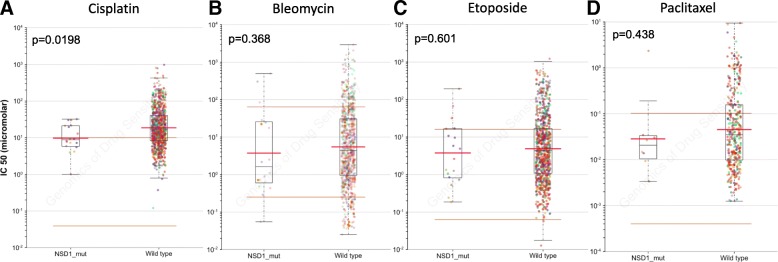
Cells with NSD1 mutations show increased sensitivity to cisplatin, but not to other DNA-damaging agents. Pan-cancer cohort showing sensitivity of cells with or without NSD1 mutations to treatment with (**a**) cisplatin (18 mutant and 798 wild-type cell lines); (**b**) bleomycin (18 mutant and 817 wild-type cell lines); (**c**) etoposide (18 mutant and 834 wild-type cell lines); and (**d**) paclitaxel (8 mutant and 377 wild-type cell lines). Adapted from the Wellcome Sanger Institute website Cancerrxgene.org

To begin exploring the factors underlying the survival differences between patients harboring tumors with wild-type and mutant NSD1, we used Cancerrxgene.org, a database in which > 1000 genetically characterized human cancer cell lines have been screened against a wide range of anti-cancer drugs [22]. Although the database did not include enough head and neck cancer cell lines for separate cohort analysis, the pan-cancer cohort showed significantly increased sensitivity (p = 0.0198) to cisplatin for cell lines with NSD1 mutations (n = 18) compared to those with wild-type NSD1 (n = 798) (Fig. 6A). Cisplatin, a DNA crosslinking agent, is the standard chemotherapy drug used as a radiation sensitizer for locally advanced HNSCC [23]. Increased sensitivity to cisplatin may in part explain why NSD1 mutations confer a survival advantage compared to wild-type NSD1 in HPV(−) tumors. In contrast to improved sensitivity to cisplatin, NSD1 mutant cell lines had the same sensitivity as NSD1 wild-type cells to several other types of DNA damaging agents: bleomycin (Fig. 6B), a radiomimetic drug that produces DNA single and double strand breaks [24]; etoposide (Fig. 6C), a topoisomerase II inhibitor that generates DNA double strand breaks (DSBs) [25, 26]; and paclitaxel (Fig. 6D), a DNA damage-inducing antimicrotubular drug.

**Fig. 7:**
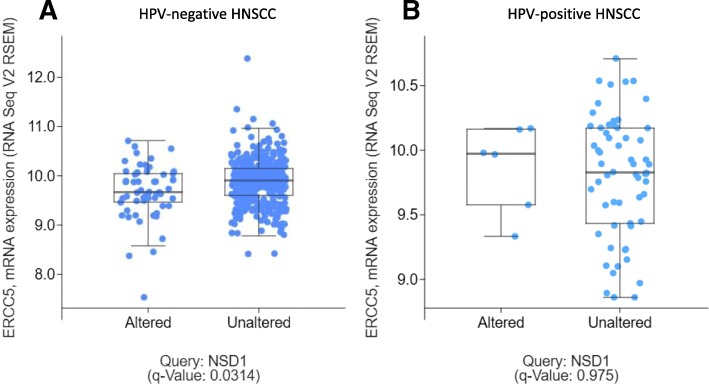
Relative to NSD1 wild-type tumors, ERCC5 has lower expression in NSD1 mutant HPV(−) tumors, but not in NSD1 mutant HPV(+) tumors. Scatterplots showing ERCC5 expression in NSD1 wild-type and mutant HPV-negative (**a**) and HPV-positive (**b**) tumors. Adapted from cBioPortal.org [41, 42]

Inactivation of ERCC5 has been shown to result in increased sensitivity to cisplatin in a variety of cancers [27–29]. The TCGA cohort was queried to determine if NSD1 mutations correlated with ERCC5 expression. Expression of ERCC5 was significantly lower in HPV(−) tumors with NSD1 mutations, compared to HPV(−) tumors with wild-type NSD1 (q = 0.0314) (Fig. 7A). However, for the limited number of HPV(+) tumors (*n* = 6 for NSD1 mutants), there was no difference in ERCC5 expression between those tumors with wild-type and mutated NSD1 (q = 0.975) (Fig. 7B).

### NSD1 knockdown increases cell sensitivity to DNA-crosslinking agents, but not to other DNA-damaging agents

**Fig. 8:**
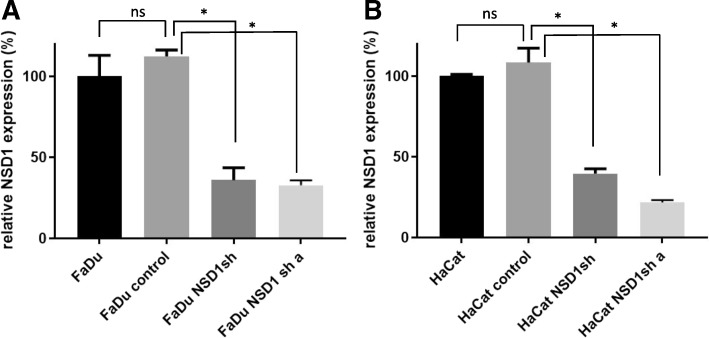
Development of NSD1-depleted cell lines. FaDu and HaCat cells were transfected with control or NSD1 shRNAs. Puromycin-resistant clones were selected. NSD1 expression was determined by RT-PCR in wild-type FaDu (**a**) and HaCat (**b**) cells or cells expressing control or NSD1 shRNA. (csh = control shRNA; NSD1sh = NSD1 shRNA knockdown)

With indications that NSD1 mutant and wild-type cells respond differently to cisplatin (Fig. 6A), we depleted NSD1 to determine the effect of NSD1 on survival of HNSCC cells or immortalized keratinocytes following treatment with platinum or other types of DNA-damaging agents. We first depleted NSD1 with specific shRNAs in FaDu, SCC35 (both are human head and neck cancer cell lines) cells, and HaCat (human immortalized, non-tumorigenic epithelial) cells, and selected knockdown clones using different shRNAs for each line that had > 50% depletion of NSD1 expression compared to parental cells or cells expressing control shRNA (Fig. 8).

**Fig. 9:**
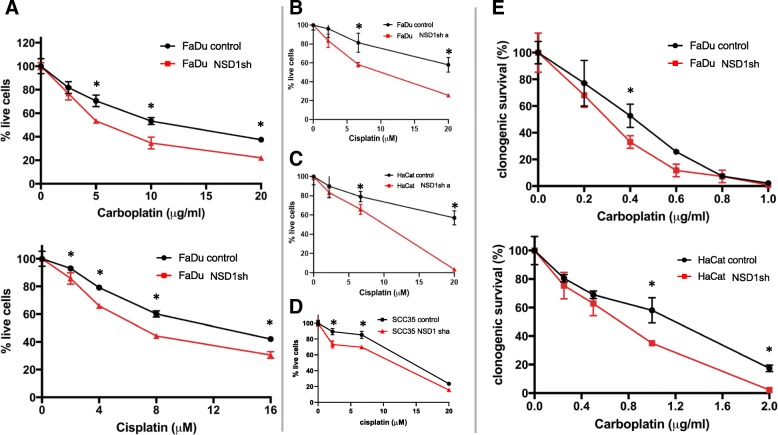
NSD1 knockdown increases cell sensitivity to DNA crosslinking agents. (**a**) Short-term viability assay using CellTiterGlo (Promega) of FaDu cells expressing control (black) or NSD1 (red, NSD1sh) shRNA, treated or not with carboplatin or cisplatin (as indicated) for 72 h. Short-term viability of FaDu (**b**), HaCat (**c**), or SCC35 (**d**) cells expressing control (black) or another NSD1 (red, NSD1sh a) shRNA, treated or not with cisplatin. (**e**) Long-term clonogenic survival of FaDu (top) and HaCat (bottom) cells expressing control (black) or NSD1 (red) shRNA, treated or not with carboplatin. * Indicates P < 0.05

Cell viability assays of NSD1 wild-type (control) and NSD1-depleted (NSD1 sh) FaDu cells showed that NSD1 knockdown rendered cells more sensitive to increasing concentrations of carboplatin and cisplatin (Fig. 9A top and bottom). FaDu, HaCat, or SCC35 cells expressing another NSD1 shRNA (NSD1 sh a) were also more sensitive to cisplatin than cells expressing wild-type NSD1 (Fig. 9B, C, and D). Clonogenic survival of FaDu and HaCat cells demonstrated that increasing concentrations of carboplatin also inhibited survival of NSD1-depleted cells (Fig. 9E). Thus, NSD1 knockdown caused increased sensitivity of epithelial cells to platinum-based chemotherapy drugs in both short-term cell viability and long-term cell survival assays.

**Fig. 10:**
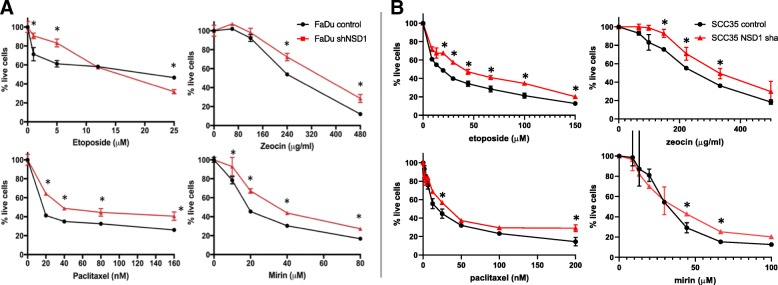
NSD1 knockdown does not increase cell sensitivity to other types of DNA-damaging agents. Short-term viability assay using CellTiterGlo (Promega) of FaDu (**a**) or SCC35 (**b**) cells expressing control (black) or NSD1 (red) shRNA, treated or not with etoposide, zeocin, paclitaxel, and mirin. * Indicates P < 0.05

As opposed to platinated drugs, cell viability assays showed that depletion of NSD1 did not sensitize cells to etoposide, zeocin (a chemical analog of radiomimetic drug bleomycin), paclitaxel, or mirin, an MRE11 inhibitor that prevents activation of a DNA damage sensor molecule Ataxia Telangiectasia Mutated (ATM) and DNA damage signaling (Fig. 10 and B). Conversely, NSD1 knockdown cells were relatively resistant to treatment with increasing concentrations of zeocin, paclitaxel, and mirin. Etoposide had a concentration-dependent effect on FaDu cells, with NSD1 knockdowns being more resistant to etoposide at low concentrations (5 μM and 10 μM), but more sensitive to etoposide at higher concentrations (20 μM and 25 μM).

**Fig. 11:**
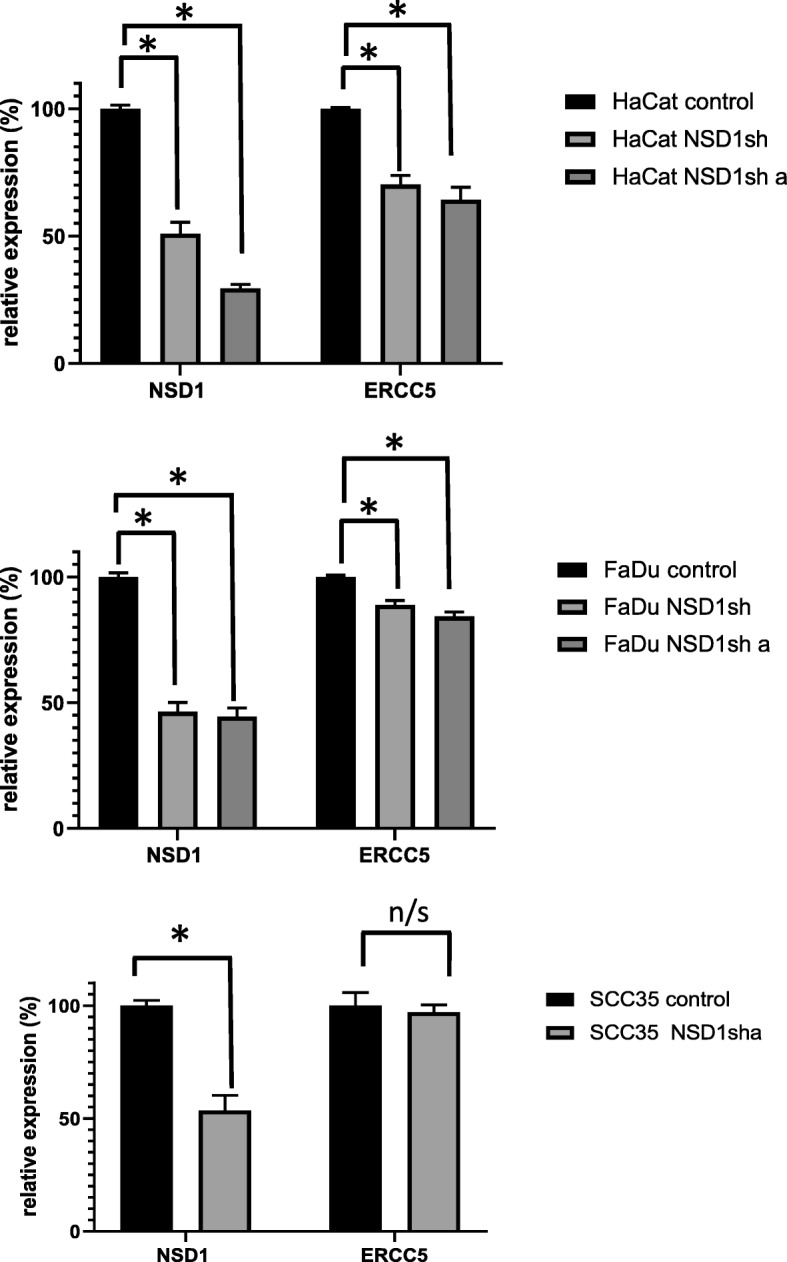
Relative expression of NSD1 and ERCC5 in HaCat (top), Fadu (middle), and SCC35 (bottom) cells expressing control or NSD1 shRNAs as determined by RT-PCR

Expression of ERCC5 was decreased in HPV(−) tumors with NSD1 mutations, compared to HPV(−) tumors with wild-type NSD1 (Fig. 7A). ERCC5 mRNA levels were also lower in HaCat and FaDu, but not in SCC35 cells expressing NSD1 shRNAs, as compared to control shRNA expressing cells (Fig. 11).

## Discussion

A histone H3 lysine 36 (H3K36) methyltransferase and chromatin modifier, NSD1 plays a role in epigenetic DNA regulation and is essential in normal human development [30]. NSD1 is also a powerful transcriptional co-factor with the ability to repress and activate expression of many genes [31]. Germline inactivating mutations in NSD1 are responsible for Sotos syndrome, a childhood overgrowth condition in which patients are at increased risk of cancer and display an NSD1 mutation-specific DNA methylation signature [32–34]. Loss-of-function mutations in NSD1 are associated with global genome hypomethylation in head and neck cancer [15, 16]. Inactivating NSD1 mutations have also been described in several other malignancies, including lung cancer, clear cell renal carcinoma, and skin cancer, suggesting a role of NSD1 as a tumor suppressor [35–38]. In contrast, in acute myeloid leukemia, a recurring translocation that fuses NSD1 to nucleoporin-98 (NUP98) leads to leukemogenesis through dysregulated H3K36 methylation at certain genetic loci [39].

Alterations in NSD1 expression have already shown promise as a prognostic biomarker in various cancers. In neuroblastomas and gliomas, NSD1 promoter methylation-associated gene silencing predicts worse survival [40]. In laryngeal cancers, loss-of-function mutations in NSD1 were shown to be a favorable prognostic biomarker [17]. While NSD1 mutations were also present in oral, oropharyngeal, and hypopharyngeal cancers, NSD1 mutations at non-laryngeal sites were not associated with better overall survival or recurrence-free survival, suggesting molecular differences between anatomical subsites [17].

Unique epidemiological, molecular, biological and clinical differences have led to the increasing recognition of HPV-positive head and neck cancer as distinct from HPV-negative HNSCC. Our data examined NSD1 mutations in both HPV(+) and HPV(−) tumors in the expanded TCGA HNSCC dataset and identified a subgroup of inactivating NSD1 mutations in both the HPV(+) and HPV(−) cohorts (Figs. 1 and 2). NSD1 mutations correlated with improved survival in HPV(−) tumors (Fig. 5 A), consistent with previously published work in laryngeal tumors [17, 20]. The worse survival of HPV(−) patients with NSD1 wild-type tumors did not correlate with worse clinical characteristics; in fact these patients smoked less than the HPV(−) patients with NSD1 mutations (Table 1). While this manuscript was under revision, another study demonstrated similar results with the frequency of NSD1 mutations in HPV-negative heavy smokers (> 20 pack year) of 18.5%, 4.55% in HPV-negative light smokers (≤ 20 pack year), and 2.22% in HPV-negative never smokers [43].

Interestingly, NSD1 mutations were associated with decreased survival in patients with HPV(+) HNSCCs (Fig. 5 B). No differences in clinical characteristics were found that could explain the survival difference between HPV(+) patients with and without NSD1 mutations (Table 2). Although the TCGA is the largest molecularly characterized cohort of HPV(+) HNSCC publicly available, these statistically significant survival results are based on a small number of NSD1 mutant tumors that are HPV(+) (n = 6) and require further validation in a separate larger cohort.

NSD1 mutations were associated with differences in gene expression of ERCC5 based on HPV status (Fig. 7). We demonstrated that ERCC5 expression was lower in HPV(−) but not HPV(+) tumors that harbored NSD1 mutations (compared to HPV(−) or HPV(+) NSD1 wild-types, respectively) (Fig. 7). Moreover, ERCC5 mRNA levels were lower in two of three NSD1 shRNA expressing cell lines tested (Fig. 11). ERCC5 is a single strand-specific nuclease that participates in nucleotide excision repair. Inactivation of ERCC5 has been shown to result in increased sensitivity to cisplatin in a variety of cancers, including non-small cell lung cancer, epithelial ovarian cancer, and uroepithelial cancer [27–29]. Given the strong involvement of ERCC5 in response to cisplatin treatment, whether or not lower expression of ERCC5 contributes to the increased cisplatin sensitivity of NSD1 mutant HPV(−) HNSCCs deserves further investigation. Given opposite correlation of NSD1 mutations with survival and ERCC5 expression in HPV(+) versus HPV(−) HNSCC, the concept that NSD1 may play different roles in epigenetic DNA regulation based on HPV status should be additionally explored.

Using isogenic cell lines, our data showed that even partial downregulation of NSD1 (25–50%, Figs. 8 and 11) sensitized HPV(−) HNSCC cells and immortalized keratinocytes to cisplatin and carboplatin (Fig. 9), which may, at least in part, explain the survival advantage of NSD1 mutations in HPV(−) HNSCC (Fig. 5 A). In contrast to platinated compounds, FaDu and SCC35 cells with shRNA-mediated depletion of NSD1 became somewhat more resistant to several DNA damaging drugs, including zeocin, etoposide and paclitaxel (Fig. 10), while available databases of various cancer cell lines demonstrated that NSD1 mutation status did not alter response to the same treatment (Fig. 6 B). The most likely explanation for that is a partial NSD1 depletion in isogenic cells in our experiments versus 100% NSD1 enzymatic inactivation due to mutation in cells derived from different origins of cancer.

The improved treatment response of NSD1 knockdown was limited to the DNA crosslinking agents cisplatin and carboplatin and was not seen with other types of DNA damaging agents tested: etoposide (topoisomerase II inhibitor), zeocin (radiomimetic), paclitaxel (microtubule inhibitor, also used in head and neck cancer treatment), and mirin (MRE11 inhibitor). Together, these data suggest that platinum-based chemotherapy better targets HPV(−) tumors with NSD1 mutations, while those without NSD1 mutations (which also have a worse prognosis) may require alternative treatments.

## Conclusions

In this study, we identify in the expanded TCGA HNSCC database a subgroup of inactivating NSD1 mutations in both the HPV(+) and HPV(−) cohorts. NSD1 mutations appear to be associated with opposite survival effects in HPV(+) and HPV(−) tumors, with NSD1 mutations correlating with improved survival in HPV(−) tumors but worse survival in HPV(+) tumors. However, the prognostic role of NSD1 mutations in HPV(+) tumors must currently be taken with caution, as it has yet to be examined in a larger cohort. In HPV(−) tumors, the survival advantage of NSD1 mutations can in part be explained by increased sensitivity to the DNA-crosslinking agents cisplatin and carboplatin.

## Data Availability

This review article is original and has not been published elsewhere.

## Abbreviations

ATM: Ataxia-telangiectasia mutated serine/threonine kinase
csh: Control shRNA
DSB: Double strand breaks
HNSCC: Head and neck squamous cell carcinoma
HPV: Human papillomavirus
NSD1: Nuclear receptor-binding SET domain protein 1
NSD1sh: NSD1 shRNA knockdown
NUP98: Nucleoporin-98
OPSCC: Oropharyngeal squamous cell carcinoma
TCGA: The Cancer Genome Atlas
